# Rosin-Modified Microcrystalline Cellulose for Enhancing Polylactic Acid-Based Composites with Good Interfacial Compatibility and Mechanical Performance

**DOI:** 10.3390/polym18070889

**Published:** 2026-04-06

**Authors:** Fuquan Zhao, Xiaoyu Xie, Yu Meng, Lijia Wang, Zilin Zhu, Lingqing Chen, Lijie Jiang, Xiaofan Zhou, Ming Yan

**Affiliations:** 1 Jiangsu Co-Innovation Center of Efficient Processing and Utilization of Forest Resources, Jiangsu Provincial Key Lab of Pulp and Paper Science and Technology, College of Light Industry and Food, National Engineering Research Center of Biomaterials, Jiangsu Engineering Research Center of Bamboo and Wood Carbon·Fixation Materials and Structures, Nanjing Forestry University, Nanjing 210037, China; 19852851268@163.com (F.Z.); 501750346@njfu.edu.cn (X.X.); azzzl@njfu.edu.cn (Z.Z.);; 2 Zhejiang Jinchang Special Paper Co., Ltd., Quzhou 324000, China; mengdreamyu@126.com (Y.M.); abcsok2025@163.com (L.W.); 3 Sherwin-Williams South China Technology Center Co., Ltd., Foshan 528306, China; lingqing.chen@sherwin.com; 4 Guangdong Paper Industry Research Institute Co., Ltd., Guangzhou 510300, China; m18920606035@126.com

**Keywords:** microcrystalline cellulose, rosin emulsion, polylactic acid, screw extrusion, interfacial compatibility

## Abstract

The interfacial incompatibility and insufficient mechanical performance of polylactic acid (PLA)/cellulose composites have severely restricted their practical applications. To address the critical issue of interfacial incompatibility in PLA/cellulose composites, this work developed a novel strategy employing rosin emulsion for blending modification of microcrystalline cellulose (MCC), followed by a one-step extrusion process to fabricate PLA composites. The corresponding analyses confirmed that the rosin has been successfully added to MCC surfaces, forming the hydrophobic interface while maintaining the cellulose I crystalline structure. Subsequently, rosin emulsion-modified MCC (MCC-R) reinforced PLA (PLA/MCC-R) composites were fabricated via twin-screw extrusion at varying MCC-R contents. The testing results illustrated that the introduction of 8 wt% MCC-R can enhance the mechanical properties of PLA/MCC-R composites with the flexural strength (125.5 MPa), tensile strength (30.8 MPa), Young’s modulus (1.19 GPa), and elongation at break (3.07%), which was attributed to enhanced filler dispersion and interfacial stress transfer. Overall, this work established a facile and sustainable strategy for developing multifunctional PLA composites with engineered interfaces and mechanical robustness, which is vital for practical application. The as-prepared PLA composites show promising application prospects in environmentally friendly packaging, biodegradable disposable products, and lightweight structural components.

## 1. Introduction

The widespread use of petroleum-based plastics in packaging, agriculture, healthcare, and industrial applications stems from excellent physicochemical properties [1,2]. However, the non-degradability of traditional petroleum-based raw materials has raised significant environmental concerns due to persistent pollution and unsustainable resource utilization [3,4]. Emerging as one of the most promising bio-based polymers, poly(lactic acid) (PLA) possesses a unique combination of high stiffness, optical clarity, processability, and complete compostability [5,6,7,8,9]. However, its commercial adoption faces limitations [10], which is due to inherent weaknesses such as molecular chain rigidity, poor crystallization kinetics, low thermal stability, and unsatisfactory barrier properties against water vapor and oxygen [11,12,13,14]. These drawbacks have restricted its extensive applications in packaging, biomedicine, smart, and energy storage.

In seeking solutions to these limitations of PLA materials, researchers have increasingly focused on cellulose-reinforced PLA composites [15]. Cellulose, a linear biopolymer composed of β-1,4-glycosidic linked glucose units (C_6_H_10_O_5_)_n_ [16]. It offers exceptional potential as a reinforcing filler for PLA composites due to its impressive mechanical strength, low density, renewability, and complete biodegradability [17,18]. Thus far, various processing methods have been explored to incorporate cellulose into the PLA matrix, ranging from solution casting to melt injection molding [1] and extrusion techniques [19]. Effective processing design is critical for achieving ideal filler dispersion and interfacial adhesion in composite preparation [20], among these, twin-screw extrusion has gained particular attention as an environmentally friendly processing route due to its energy efficiency and minimal solvent requirements [21]. Appropriate processing and post-treatment can effectively regulate the crystallization and interfacial structure of composites without changing material components [22,23]. However, the successful integration of MCC into PLA faces a fundamental compatibility challenge: the hydrophilic nature of cellulose creates poor interfacial adhesion with the hydrophobic PLA matrix, often resulting in suboptimal mechanical performance [24]. This has driven the development of various surface modification strategies of cellulose, including esterification [25], silylation [26], and polymer grafting approaches [27], which aim at improving the dispersibility and interfacial bonding of cellulose into the PLA matrix. However, all of these cellulose surface modifications need complicated pretreatments or consume too much time. Furthermore, cellulose surface modifications will alter the natural structure and physicochemical characteristics. Therefore, the addition of compatibilizers has been considered one of the most effective ways to improve the dispersibility and interfacial compatibility between cellulose and PLA. Based on this, it is necessary to find green and efficient compatibilizers to improve the interfacial compatibility between cellulose and PLA.

Notably, recent work has demonstrated that rosin modification can simultaneously enhance the hydrophobicity of cellulose while imparting additional functional properties to the composite material [28]. Rosin, a natural resin derived from pine trees, presents unique advantages for cellulose modification due to its inherent hydrophobicity and biocompatibility [29,30]. These excellent properties have established rosin as a valuable material in diverse industrial applications ranging from paper sizing to coating formulations [10,31]. Recently, Castro et al. [32] reported the successful hydrophobization of cellulose nanocrystals (CNC) via esterification with rosin derivatives. The CNC modified in this way exhibited notable antibacterial activity against Gram-negative pathogens, which is attributed to the inherent antimicrobial components and enhanced surface compatibility of rosin. Niu et al. [16] conducted rosin-based modification of cellulose nanofibers (CNF) and subsequently employed them as reinforcing fillers in a PLA matrix. The mechanical properties of the obtained film have been improved. While these results are promising, conventional modification methods typically require energy-intensive processing conditions, including high reaction temperatures and prolonged reaction times under inert atmospheres—limitations stemming from the steric hindrance effects of rosin’s hydrogenated phenanthrene ring structure [26]. These demanding processing requirements, coupled with the high cost of nanocellulose substrates, have posed significant barriers to the scalable production of rosin-modified cellulose composites [29]. Therefore, rosin should be used as a compatibilizer to improve the interfacial compatibility between cellulose and PLA by employing a twin-screw extruder. Unfortunately, to the best of our knowledge, there are few relevant studies at present.

In this context, an innovative processing strategy that addresses several key challenges, including interfacial incompatibility and insufficient mechanical strength, is needed in the development of high-performance PLA composites. Herein, this work innovatively proposes a twin-screw extrusion strategy to modify MCC by introducing the rosin emulsion and fabricate multifunctional PLA-based composites. Departing from conventional complex chemical treatments, the approach integrates the hydrophobic modification and in-situ composite fabrication in the extrusion step, thus enhancing the dispersibility and interfacial compatibility of MCC in the PLA matrix. Structural characterizations, including FTIR, XRD, and ESEM, confirm the successful incorporation of rosin into MCC without disrupting its crystalline structure. Compared with the raw material, the resulting PLA/MCC-R composite possesses good mechanical properties and thermal processing performance. In a word, this eco-friendly method proposed in this work not only can improve the mechanical robustness and thermal processing performance of PLA composites but also offers a scalable, sustainable route for developing biomass-based materials, which would hold a promise for biodegradable packaging applications to replace petroleum-based plastics.

## 2. Materials and Methods

### 2.1. Materials

The MCC was procured from Shanghai Aladdin Bio-Chem Technology Co., Ltd., Shanghai, China. It appeared as a white or off-white powder with a particle size of 100–200 mesh. The rosin-based nanoemulsion (approximately 30 wt% rosin content, pH 5–6) was procured from Taishun Chemical Products Trading Company, Jinshui District, Zhengzhou City, China. PLA was purchased from Hongda New Materials (USA4032D). The weight-average molecular weight (Mw) and density of PLA were approximately 3 × 10^4^ g/mol and 1.3 g/cm^3^, respectively, with a melting temperature range of 160–200 °C. All materials were utilized without supplementary purification steps. Before the experiment, MCC and PLA were dried in a blast drying oven (Shanghai Yiheng Scientific Instrument Co., Ltd., Shanghai, China) for 24 h at 70 °C and 60 °C, respectively.

### 2.2. Modification of MCC by Using Rosin Emulsion

Firstly, 10 g of rosin emulsion was mixed with 30 g of MCC to achieve a mass ratio of rosin to MCC of 1:10. Subsequently, the MCC/rosin composite was prepared by employing a twin-screw extruder (CZR 2014, Wanbiao General Equipment Co., Ltd., Beijing, China) (3 cycles, 100 °C, 50 rpm). The obtained composite was defined as MCC-R. Before melt blending with PLA, the MCC-R composite was vacuum-dried at 80 °C for 24 h. The moisture content was measured to be 0.21 ± 0.03 wt%, which ensures stable extrusion quality and avoids adverse effects on PLA hydrolysis and processing.

### 2.3. Preparation of MCC-R Reinforced PLA Composite Specimens

PLA particles were mixed with MCC-R at various contents (0, 2, 5, 8, and 10 wt%), which were selected based on relevant literature and preliminary experiments to achieve effective reinforcement while avoiding filler agglomeration [16]. The mixtures were then melt-blended in a twin-screw extruder at 180 °C with a rotating speed of 50 rpm for 3 cycles to ensure uniform dispersion of the filler in the PLA matrix. The obtained samples were named PLA/XMCC-R, where X was the percentage of MCC-R. Subsequently, the injection-molded specimens of PLA/XMCC-R were prepared by using an SZS-20 injection molding system (Wuhan Ruiming Experimental Instrument Manufacturing Co., Ltd., Wuhan, China). The temperature of the mold plate area and the injection area were 90 °C and 180 °C, respectively. The first and second injection pressures were both set at 0.5 MPa. The mold clamping time, first injection time, and second injection time were 4 s, 3 s, and 5 s, respectively.

### 2.4. Characterizations

#### 2.4.1. Environmental Scanning Electron Microscope (ESEM)

Surface morphologies were analyzed using a Quanta 200 environmental scanning electron microscope (ESEM, FEI Company, Hillsboro, OR, USA). All samples were sputter-coated with gold prior to observation, and the characterization was performed at an accelerating voltage of 15 kV.

#### 2.4.2. Fourier Transform Infrared Spectroscopy (FTIR)

A Vertex 80V Fourier transform infrared (FTIR) spectrometer (Bruker Corporation, Billerica, MA, USA) was utilized to characterize the samples. After pressing the samples into KBr tablets, the mid-infrared transmission mode was selected with a scanning wavelength range of 400~4000 cm^−1^, a resolution of 4 cm^−1^, and 32 scans per spectrum.

#### 2.4.3. X-Ray Diffraction (XRD)

The crystallinity of MCC and MCC-R was analyzed using an X-ray diffractometer (Rigaku Corporation, Tokyo, Japan) with a scanning range of 5~50°at a rate of 10°/min. The crystallinity index (*CrI*) was calculated using the following formula [33].

$$CrI=\left(I_{002}-I_{am}\right)/I_{002}\times 100\%$$

*CrI* denotes the relative crystallinity, with *Iam* representing the diffraction intensity of the amorphous phase and *I*_002_ signifying the peak diffraction intensity of the (002) crystalline plane.

#### 2.4.4. Thermogravimetric Analysis (TGA)

Five to ten milligrams of the sample were accurately weighed and placed in an alumina crucible for thermogravimetric characterization using a 209F1 thermogravimetric analyzer (Netzsch Gerätebau GmbH, Selb, Germany). The N_2_ gas flow rate was maintained at 50 mL/min during thermal analysis, which was performed over a temperature range of 30~600 °C with a heating rate of 10 °C/min.

#### 2.4.5. Differential Scanning Calorimetry (DSC)

The glass transition temperature (T_g_) of MCC-R reinforced PLA was measured using a 214 F1 Phoenix differential scanning calorimeter (DSC, Netzsch Gerätebau GmbH, Selb, Germany). A heat–cool–heat procedure was employed to eliminate thermal history and ensure accurate Tg determination. Samples were first heated from 0 °C to 200 °C at 10 °C/min, then cooled to 0 °C at 10 °C/min, and reheated to 200 °C at 10 °C/min under nitrogen protection. The Tg was obtained from the second heating scan.

#### 2.4.6. Mechanical Property

The three-point bending test on specimens was carried out with a UTM6503 universal testing machine (MTS EXCEED E44, Mester Industrial System Co., Ltd., Shanghai, China). The tests were performed following ASTM D 790. Specimen dimensions were configured as 64 mm (length) × 10 mm (width) × 4 mm (thickness). The test parameters included a 64 mm span and a crosshead speed set to 3 mm/min. Each specimen underwent testing five times, and the average result was documented. For the tensile test, the identical universal testing machine was employed, with a crosshead speed of 5 mm/min. Tensile testing specimens were prepared with dimensions of 75 mm (length) × 5 mm (width) × 2 mm (thickness). Tensile strength, tensile modulus, and elongation at break were measured for each sample.

## 3. Results

### 3.1. Blending Modification of MCC with Rosin

**Figure 1:**
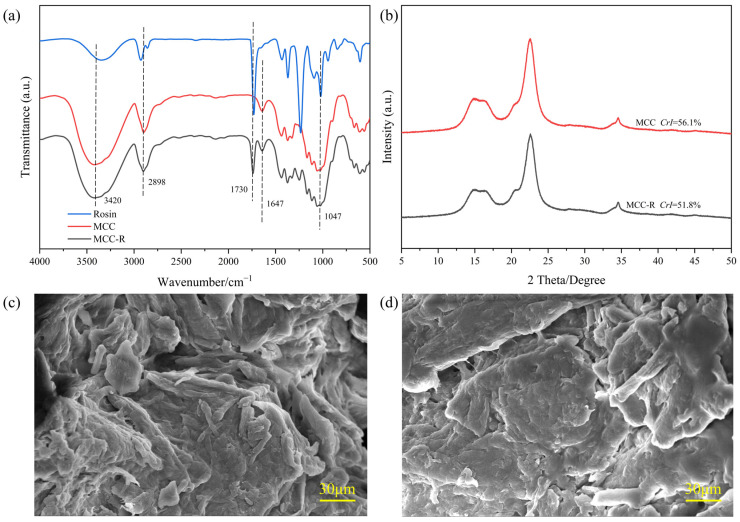
(**a**) FTIR spectra and (**b**) X-ray diffractograms of MCC and MCC-R. ESEM images of (**c**) MCC and (**d**) MCC-R.

The successful blending modification of MCC with rosin was systematically verified through comprehensive spectroscopic, crystallographic, and ESEM analyses. As shown in Figure 1a, FTIR spectra of both MCC and MCC-R exhibit characteristic cellulose absorption bands at 3420 cm^−1^ (O-H stretching vibration), 2889 cm^−1^ (C-H symmetric stretching), 1647 cm^−1^ (H-O-H bending of adsorbed water molecules), and 1047 cm^−1^ (C-O-C pyranose ring vibration) [32]. Crucially, the emergence of a new absorption band at 1730 cm^−1^ in MCC-R, corresponding to the carbonyl stretching vibration of rosin esters [16], provides direct evidence of successful blending modification. Notably, the relatively unchanged hydroxyl stretching band (3340 cm^−1^) suggests that rosin modification primarily occurs on the MCC surface rather than chemical modification, preserving the fundamental cellulose structure while introducing hydrophobic functionality. Furthermore, complementary XRD analysis (Figure 1b) reveals that both MCC and MCC-R maintain the characteristic diffraction pattern of cellulose I with prominent peaks at 2θ = 14.9°, 22.5°, and 34.5°, which is corresponding to the (110), (200), and (004) crystallographic planes, respectively [32]. It can be observed from Figure 1b that a modest decrease in crystallinity index from 56.1% ± 0.2% for pristine MCC to 51.8% ± 0.1% for MCC-R, which indicates partial disruption of the native hydrogen bonding network during blending modification. Importantly, these results confirm that the rosin modification process does not significantly alter the fundamental cellulose crystal structures [29]. Combined with the introduction of hydrophobic rosin moieties, this structural preservation suggests an optimal balance between surface functionality modification and bulk property retention. This is a critical prerequisite for the practical applications of the resulting polymer composites.

The ESEM images of MCC and MCC-R are shown in Figure 1c,d. MCC displays characteristic fibrous morphology with rough and porous surfaces, reflecting the natural arrangement of cellulose microfibrils [34]. After the blending modification by using rosin, MCC-R shows significant morphological alterations, which include reduced surface roughness, partial pore filling, and an overall more compact structure. These phenomena confirm the successful blending of rosin on MCC. From a microstructural evolution perspective, rosin likely interacts with MCC during molten blending and then infiltrates the porous MCC structure during extrusion. This blending modification effectively alters the surface characteristics of MCC while preserving its natural structure. Collectively, these morphological changes have significant implications for the performance of composite materials. A smoother and more compact surface morphology enhances interfacial adhesion with hydrophobic PLA matrices, whereas the reduction in porosity diminishes the tendency for moisture absorption. These phenomena are consistent with the findings from the FTIR and XRD analyses, thereby confirming the efficacy of the extrusion-based modification method. This approach realizes the controlled surface modification of MCC without sacrificing the native cellulose structure, thereby laying a solid foundation for the development of high-performance composites.

### 3.2. Infrared Spectroscopy and Morphology of PLA/MCC-R Composites

**Figure 2:**
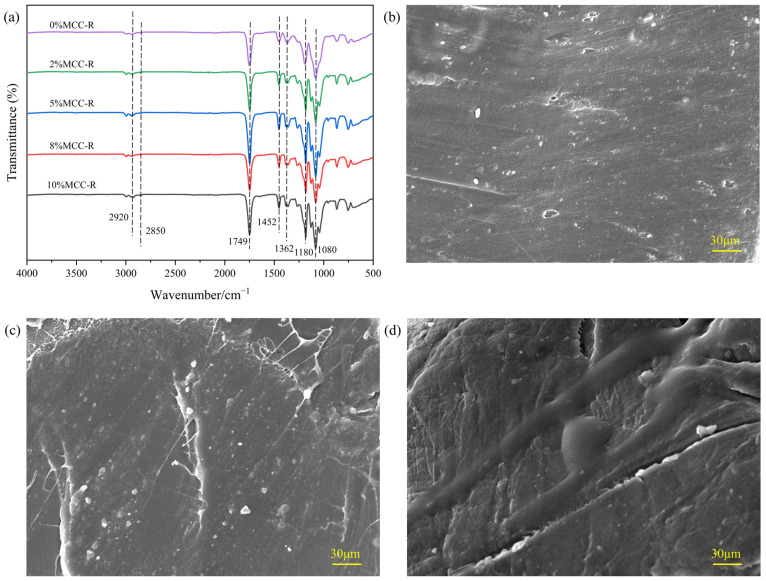

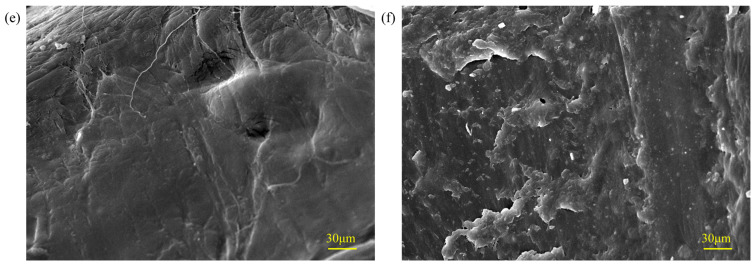
(**a**) FTIR spectra of PLA reinforced with different contents of MCC-R. ESEM images of PLA reinforced with (**b**) 0%MCC-R, (**c**) 2%MCC-R, (**d**) 5%MCC-R, (**e**) 8%MCC-R, and (**f**) 10%MCC-R.

Figure 2a compares the FTIR spectra of neat PLA and PLA/MCC-R composites prepared by using a twin-screw extrusion. It can be found that the characteristic absorption bands of PLA appear at 2920 cm^−1^ and 2850 cm^−1^, which arise from asymmetric CH_2_ stretching and symmetric CH_2_ stretching, respectively. Moreover, the strong peak at 1749 cm^−1^ corresponds to carbonyl (C=O) stretching of the ester group, while the band at 1180 cm^−1^ arises from C-O stretching vibrations [35,36]. The characteristic peaks at 1452 cm^−1^ and 1362 cm^−1^ arise from CH_3_ deformation vibrations, while the characteristic peak at 1080 cm^−1^ corresponds to C-O-C stretching [37]. Importantly, all PLA/MCC-R composites exhibit identical peak positions and shapes to neat PLA. This result indicates that no chemical reactions occurred during processing and that MCC-R interacts with PLA only through physical blending. In a word, the extrusion process proposed in this work can achieve uniform physical blending of MCC-R and PLA while preserving the chemical integrity of both components. The hydrophobic rosin layer effectively improved interfacial compatibility between MCC-R and PLA, which is crucial for developing high-performance biocomposites. The morphological evolution of the surface morphology of PLA/MCC-R composites with increasing filler content is shown in Figure 2b–f. It can be observed from Figure 2 that the surface of pure PLA displays a characteristically smooth microstructure, which is consistent with the other work [38]. When the content of MCC-R is 2%, the surface of the PLA/MCC-R composite begins to show fine textures. MCC-R can not be uniformly covered on the surface of the PLA/MCC-R composite due to its low filler content. Optimal morphology is achieved at 5–8 wt% MCC-R, where the surfaces exhibit homogeneous roughness, indicating good filler distribution and matrix-filler adhesion. However, it can be observed that the excessive MCC-R content would lead to particle agglomeration and surface roughening as the content of MCC-R increases to 10%. It might be due to that the interfacial adhesion between PLA and MCC-R becomes insufficient at high filler content [39]. Overall, it can be concluded that the 8 wt% dosage of MCC-R appears to represent the percolation threshold for this composite system, beyond which excessive filler disrupts the matrix continuity and compromises material integrity.

As the MCC-R dosage increases to 5%, the microstructure of the sample improves, with a relatively uniform surface, indicating uniform dispersion of MCC-R in PLA. The best composite effect is achieved at an 8% dosage.

### 3.3. Mechanical Properties and Thermal Performance of PLA/MCC-R Composites

**Figure 3:**
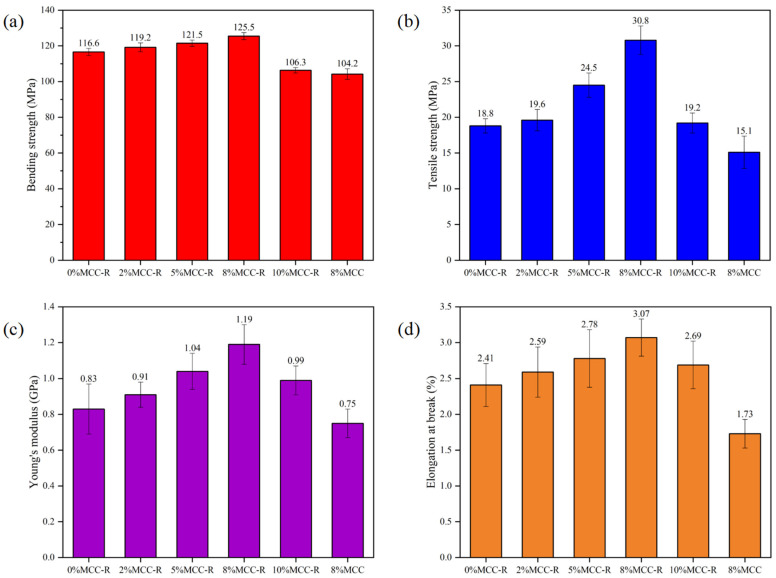
(**a**) Flexural strength, (**b**) Tensile strength, (**c**) Young’s modulus, and (**d**) Elongation at the break of PLA/MCC-R composites with different MCC-R loadings.

As illustrated in Figure 3, the mechanical performance of PLA/MCC-R composites exhibits a clear dependence on the filler content. Analysis of flexural properties reveals a progressive enhancement in strength with the content of MCC-R. As the content of MCC-R increases to 8 wt%, the flexural strength of the PLA/MCC-R composite can reach an optimal value of 125.5 MPa. Beyond this critical loading threshold, the flexural strength of the PLA/MCC-R composite would decrease to 106.3 MPa as the content of MCC-R increased to 10 wt%. Similar trends are observed for other mechanical parameters, it can be found from Figure 3b–d that the tensile strength, Young’s modulus, and elongation at break of the PLA/MCC-R composite with the 8 wt% MCC-R loading can reach up to 30.8 MPa, 1.19 GPa, and 3.07%, respectively. At lower loadings of MCC-R (2~5 wt%), the reinforcing mechanism dominates, thus leading to the gradual enhancement of mechanical properties. The peak mechanical performances at 8 wt% represent an optimal balance between these factors, where sufficient MCC-R content exists to reinforce the matrix without compromising interfacial adhesion [40]. However, exceeding this optimal loading (8 wt% MCC-R) would trigger a reversal in mechanical performance across all measured parameters. This deterioration correlates directly with the morphological changes observed in the ESEM analysis (Figure 2), where MCC-R agglomeration becomes evident at higher loadings. The aggregates act as defects that not only reduce effective stress transfer but also initiate premature failure through void formation at the filler-matrix interface [41]. These findings demonstrate that the introduction of MCC-R can successfully enhance the mechanical properties of PLA composites. However, the control of filler content of MCC-R is essential to maintain the dispersion quality and achieve optimal mechanical performance of PLA composites. Overall, the 8 wt% loading of MCC-R emerges as the most favorable composition for PLA composites, which can provide a balanced improvement in stiffness, strength, and ductility without inducing filler agglomeration. Moreover, to validate the effectiveness of rosin blending modification in PLA/MCC composites, the mechanical properties of the PLA/MCC composite (used as a reference sample and prepared under the same processing conditions) have been evaluated. It can be found that the flexural strength, tensile strength, Young’s modulus, and elongation at break of the PLA/8%MCC composite are 104.2 MPa, 15.1 MPa, 0.75 GPa, and 1.73%. The mechanical properties of the PLA/MCC composite are much lower than the PLA/8%MCC-R composite (the flexural strength, tensile strength, Young’s modulus, and elongation at break for 125.5 MPa, 30.8 MPa, 1.19 GPa, and 3.07%, respectively). Combined with the ESEM analysis, it can be concluded that the blending modification of MCC by using rosin can improve the compatibility with the PLA matrix, thus enhancing the mechanical properties of PLA/cellulose composites.

**Figure 4:**
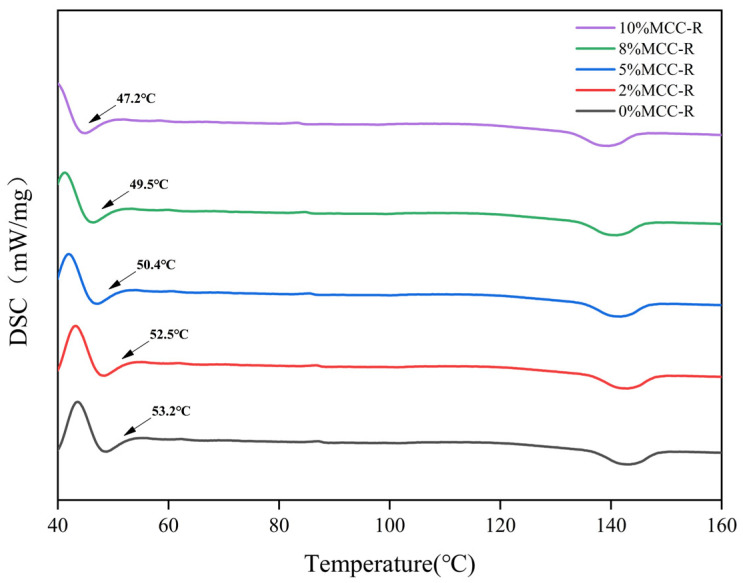
DSC curves of PLA/MCC-R composites.

**Figure 5:**
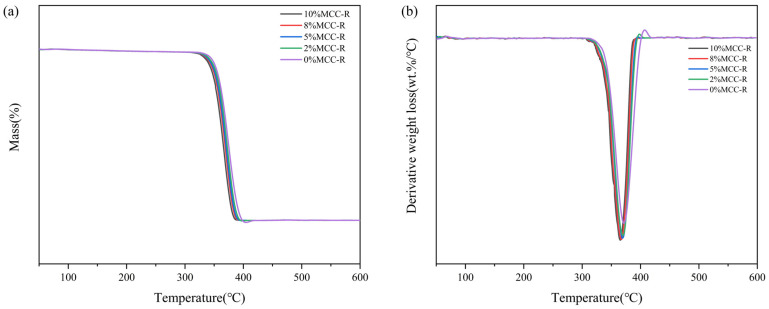
(**a**) TG curves, (**b**) DTG curves of PLA/MCC-R composites.

The thermal behavior of PLA/MCC-R composites was systematically investigated by differential scanning calorimetry (DSC) and thermogravimetric analysis (TGA), with the DSC results presented in Figure 4 and the TG/DTG results shown in Figure 5. DSC thermograms reveal a progressive decrease in glass transition temperature (T_g_) from 53.2 °C for neat PLA to 47.2 °C for the PLA/10%MCC-R composite. This depression in T_g_ can be attributed to the combined effects of MCC-R incorporation [42]. On the one hand, the cellulose component introduces additional amorphous regions, thus disrupting the regularity of PLA molecular chains. On the other hand, the rosin component can act as an interfacial lubricant, thereby enhancing the segmental mobility of PLA chains near the filler-matrix interface. These dual mechanisms effectively lower the energy barrier for chain mobility, which would result in the observed T_g_ reduction of PLA/MCC-R composites. Figure 5a,b present the TG and DTG curves, with detailed thermal stability parameters listed in Table 1. Among all samples, the PLA/8%MCC-R composite shows the highest T5% value, indicating the best initial thermal stability. While a slight downward trend in Tmax is observable with increasing MCC-R content, the differences fall within experimental error margins (Table 1). It indicates that the incorporation of MCC-R cannot significantly alter the thermal degradation behavior of PLA. The residual char yield at 600 °C gradually rises with increasing MCC-R content, which is ascribed to the carbon-rich structure of cellulose. It can be found that the above results on T_g_ and decomposition temperature highlight the influence of MCC-R on the different thermal behavior of PLA/MCC-R composites. While it significantly affects the glass transition through physical interactions with PLA chains, it has minimal impact on the chemical stability of the polymer backbone. The thermal behavior of PLA/MCC-R composites prepared in this work is favorable for practical applications, as it combines the good heat processability with the maintained thermal stability.

## 4. Conclusions

In this work, a green and efficient twin-screw extrusion strategy was developed to fabricate rosin-modified microcrystalline cellulose (MCC-R)/polylactic acid (PLA) composites with enhanced interfacial compatibility and mechanical performance. Structural characterization through FTIR, XRD, and ESEM confirmed the successful physical coating of rosin onto MCC surfaces while preserving the native cellulose I crystalline structure. The optimized composite containing 8 wt% MCC-R exhibited outstanding mechanical properties, including a flexural strength of 125.5 MPa, tensile strength of 30.8 MPa, Young’s modulus of 1.19 GPa, and elongation at break of 3.07%. These significant improvements are attributed to the enhanced filler dispersion and optimized interfacial stress transfer facilitated by the rosin modification. Thermal analysis revealed that the rosin blending modification process of MCC had a negligible impact on the decomposition behavior of PLA, maintaining excellent processing stability of PLA/MCC composites. In a word, this work establishes a practical and sustainable methodology for developing high-performance PLA composites with tailored interfaces, combining mechanical robustness with processing stability. Furthermore, the demonstrated approach can provide a viable pathway for the industrial implementation of bio-based composite materials.

## Figures and Tables

**Table 1:** Different decompositions of the corresponding temperature.

Specimens	T_5%_ (℃)	T_max_ (℃)	Degradation Stage Mass Loss (%)	Residual Yield at 600 °C (%)
0%MCC-R	320.3	372.7	96.5	0.5
2%MCC-R	318.5	370.1	95.8	1.2
5%MCC-R	317.2	369.2	95.2	1.8
8%MCC-R	325.5	367.4	94.5	2.5
10%MCC-R	315.6	365.5	93.9	3.1

## Data Availability

The original contributions presented in this study are included in the article. Further inquiries can be directed to the corresponding author.
